# Lifetime follow-up of an adult patient with pediatric-onset hypophosphatasia complicated with advanced chronic kidney disease

**DOI:** 10.1016/j.bonr.2025.101872

**Published:** 2025-08-18

**Authors:** Maria Sääf, Sigridur Björnsdottir, Mathias Haarhaus, Ellen-Margrethe Hauge, Diana Atanasova, Per Magnusson

**Affiliations:** a Department of Molecular Medicine and Surgery, Karolinska Institutet, SE-17177 Stockholm, Sweden; b Division of Renal Medicine, Department of Clinical Science, Intervention and Technology, Karolinska Institutet, Karolinska University Hospital, SE-14186 Stockholm, Sweden; c Diaverum AB, Hyllie Boulevard 53, SE-21537 Malmö, Sweden; d Department of Rheumatology, Aarhus University Hospital, Department of Clinical Medicine, Aarhus University, DK-8200 Aarhus N, Denmark; e Department of Clinical Chemistry, and Department of Biomedical and Clinical Sciences, Linköping University, SE-58185 Linköping, Sweden

**Keywords:** Alkaline phosphatase, Chronic kidney disease, Hypophosphatasia, Teriparatide, Transplantation

## Abstract

Hypophosphatasia (HPP) is a rare inborn-error-of-metabolism caused by mutations in the *ALPL* gene, resulting in deficient activity of tissue-nonspecific alkaline phosphatase and impaired skeletal mineralization. Affected individuals have a higher prevalence of chronic kidney disease (CKD) than the general population. We report a woman who underwent craniosynostosis surgery in infancy and lost her deciduous teeth prematurely. From age 27, she experienced recurrent foot pain due to multiple metatarsal fractures. Low levels of total alkaline phosphatase (ALP) was noted at 39 years of age, and low activities for the three bone-specific ALP (BALP) isoforms B/I, B1 and B2. Genetic analysis revealed 2 missense variants in the *ALPL* gene (p.Glu191Lys and p.Gly456Arg) confirming HPP. At age 44, she developed bilateral hip fissures requiring right-sided total hip replacement. Treatment with the parathyroid hormone analogue teriparatide (20 μg/day) was initiated at age 50, leading to increased BALP isoform levels indicating improved mineralization, less bone pain, and no new fractures during 9 months of treatment, which was stopped due to hypercalcemia and hyperphosphatemia. She began peritoneal dialysis at age 55 and received a kidney transplant at age 58. At age 65, seven years post-transplantation, she remained free of new fractures and significant bone pain. This case illustrates the long-term natural history of HPP with progressive skeletal complications across decades, and highlights the potential of short-term teriparatide as a therapeutic option for symptom relief and improved mineralization. It also suggests that kidney transplantation may contribute to improved bone health in HPP with advanced CKD.

## Highlights

• Lifetime follow-up to 65 years of age of a woman with hypophosphatasia
• Clinical problems of hypophosphatasia complicated by chronic kidney disease
• Clinical improvement but decreasing kidney function during teriparatide therapy
• Increased bone alkaline phosphatase isoform activities during teriparatide therapy
• No severe bone pain or new fractures during 7 years post-kidney transplantation

## 1 Introduction

Hypophosphatasia (HPP; OMIM#: 146300, 241500, 241510) is a rare inborn-error-of-metabolism that usually features rickets in children and osteomalacia in adults and is characterized by deficient tissue-nonspecific isozyme of alkaline phosphatase (TNALP), low serum alkaline phosphatase (ALP) activities, and defective bone and tooth mineralization. The clinical expression of HPP is highly variable and is classified into six different forms: perinatal lethal, perinatal benign, infantile, childhood, adult, and odontohypophosphatasia (Whyte, 2016). The more severe forms, perinatal and infantile, are usually inherited as an autosomal recessive trait while both autosomal recessive and autosomal dominant transmission can be found in the milder forms (Whyte, 2016; Whyte et al., 2018). A wide range of distinct gene variants, about 480 as of August 2025, has been described in the global ALPL gene variant database (https://alplmutationdatabase.jku.at/) (Farman et al., 2024), and approximately 75 % of these are missense variants (amino acid substitution mutations) (Silvent et al., 2014). Data in the Global HPP Registry shows that kidney disease manifestations is present in 21.3 % of individuals with adult HPP (Dahir et al., 2022).

Four genes encode for the human ALP isozymes: tissue-nonspecific (*ALPL*), placental (*ALPP*), germ cell (*ALPG*), and the intestinal locus (*ALPI*) (Minisola et al., 2025). Because bone-specific ALP (BALP) and liver ALP are encoded by the same gene locus (*ALPL*), they are referred to as isoforms of the same TNALP isozyme. Three BALP isoforms (B/I, B1 and B2) can be quantified in human serum by high-performance liquid chromatography (HPLC) (Halling Linder et al., 2009; Haarhaus et al., 2017). The B/I (bone/intestinal) isoform, that is, on average 4 % of the total serum ALP, is not a “pure” bone peak since it co-elutes with circulating intestinal ALP and the peak is composed, on average, of 70 % bone and 30 % intestinal ALP (Magnusson et al., 2002a). The circulating levels of the BALP isoforms can vary independently during the pubertal growth spurt (Magnusson et al., 1995) and in metabolic bone disease (Haarhaus et al., 2021; Magnusson et al., 2001; Magnusson et al., 2010).

In 2012, Whyte et al. (2012) presented the first report on bone-targeted enzyme-replacement therapy with recombinant human TNALP (Strensiq® (asfotase alfa), Alexion Pharmaceuticals, Inc., AstraZeneca Rare Disease, Boston, MA, USA) in infants and young children with life-threatening perinatal or infantile HPP. In a randomized open-label controlled study, enzyme-replacement therapy for 5 years in adolescents and adults with HPP demonstrated decreased serum pyridoxal 5′-phosphate (endogenous BALP substrate) levels and improved functional abilities (Kishnani et al., 2019). As of today, approved therapeutic indications for asfotase alfa comprises the more severe forms, i.e., perinatal, infantile, and childhood HPP, but not adult HPP. However, asfotase alfa is approved in Japan for all individuals with HPP regardless the age of onset. Disadvantages with asfotase alfa involve several subcutaneous injections each week, injection site reactions, lifelong treatment, and high cost.

Beneficial treatment effects, such as less pain and healing of fractures, have been reported in some individuals with adult HPP treated with recombinant human parathyroid hormone (PTH) analog, i.e., teriparatide (TPTD; N-terminal PTH 1–34) and intact PTH 1–84 (Table 1). The clinical experience with PTH analogs is, however, limited and the responsiveness seems to vary considerably among individuals with adult HPP and may be transient (Gagnon et al., 2010; Laroche, 2012).

**Table 1:** Reported case reports of TPTD therapies in adult HPP.

Reference	Age (years) Sex	Substance Dose	Duration of therapy (months)	Effect on pain	Effect on fractures	Bone turnover markers	Co-morbidities Co-medication aBMD
Whyte et al. (2007)	56, female	TPTD 20 μg/day	16	Improved	Fracture healing improved	ALP increased	aBMD prior to TPTD:
						OC increased	LS T-score + 2.4, 2 years later +1.9
						U-NTX increased	Hip T-score + 1.1, 2 years later +1.4
Camacho et al. (2008)	75, female	TPTD 20 μg/day	24	Not reported	No new fractures	ALP increased,	LS aBMD –3.3 prior to TPTD
						BALP increased	Increased 8.6 % after TPTD.
						U-NTX increased	BMD hip unchanged.
Doshi et al. (2009)	53, female	TPTD 20 μg/day	34	Improved	Fracture healing improved	ALP not followed BALP not increased U-NTX increased	Risedronate 2.5 years previously. Primidone for seizure disorder. Before TPTD: LS T-score − 1.4 Total hip T-score − 3.0. LS BMD remained stable after TPTD and dual hip T-score was −3.2.
Gagnon et al. (2010)	53, female	TPTD 20 μg/day	13	Improved	Fracture healing improved	ALP initial increase but returned to baseline PINP initial increase U-NTX initial increase	Bone biopsy
Schalin-Jäntti et al. (2010)	56, female 64, female	PTH 1–84,100 μg/day	7 + 8 18	Improved	Fracture healing improved	ALP increased BALP increased PINP increased U-INTP increased	Two sisters. BMD before TPTD: The younger sister had LS T-score + 1.2, and FN –1.4. LS T-score decreased to −0.1. BMD was not measured in the older sister due to metallic rods.
Laroche (2012)	43, female	TPTD 20 μg/day	12	Worsened	No effect	BALP unchanged OC increased CTX increased	BMD prior to TPTD: LS T-score − 1.1. TPTD did not improve total body BMD or LS BMD.
Cundy et al. (2015)	55, male	TPTD 20 μg/day	6	Improved	No new fractures during TPTD	ALP increased BALP increased PINP increased	Hypertension since age 40 years. At 50 years, renal biopsy showing IgA nephropathy. Metatarsal fracture and osteopenia. Alendronate at age 52 years when CKD stage 4. On peritoneal dialysis 14 months later. Multiple fractures. Alendronate stopped after 21 months. TPTD when on waiting list for renal transplantation. Bone biopsied performed thrice.
Camacho et al. (2016)	68, female 53, female	TPTD 20 μg/day	24 + 8 24 + 3 + 18	Not noted	No fracture during therapy	ALP increased U-NTX increased ALP normalized BALP increased, but returned to baseline CTX increased	Polymyositis treated with prednisone 2 years and ibandronate for 7 months previously. BMD prior to TPTD: LS T-score − 1.6, FN T-score − 2.9, both stable during TPTD. BMD prior to TPTD: LS T-score − 3.9 and FN T-score − 2.6. LS BMD minor increase and FN BMD minor decrease during TPTD.
Righetti et al. (2018)	67, female	TPTD 20 μg/day	12	Improved	Improved fracture healing	ALP normalized BALP increased CTX increased	Long-term corticosteroids for tendinitis. Osteoporosis, FN T-score − 3.5. Bilateral AFF after previous alendronate therapy for 10 years.
Schmidt et al. (2019)	55, female 48, male 68, female 48, male	TPTD 20 μg/day	3 3 8 12	Not reported Not reported Not reported Not reported	Bone marrow edema resolved Improved fracture healing No clear improvement of bone marrow edema No improvement of fracture healing	ALP and BALP slightly increased ALP, BALP increased but returned to baseline after discontinuation of TPTD ALP, BALP, OC increased during TPTD ALP, BALP slight increase during TPTD but returned to baseline after discontinuation.	Infusion ibandronate twice before TPTD. Osteopenia Osteopenia Osteoporosis. Rheumatoid arthritis treated with methotrexate 10 years and denosumab 2 years previously. CKD stage 3, nephrocalcinosis. Bone biopsy
Polyzos et al. (2021)	41, male	TPTD 20 μg/day for 5 months, every other day for 28 months, every third day for 9 months, thereafter once weekly	48	Mild pain did not change	Elbow fracture after 2 months TPTD but no further fracture during therapy	ALP did not increase BALP increased slightly	7 months alendronate previously BMD increased 15 % in LS and 6 % in FN from *Z*-score − 2.8 after TPTD. Calcific periarthritis shoulder.
Warren et al. (2021)	40, female	TPTD 20 μg/day	24	Not noted	Improved fracture healing	Not reported	Bilateral AFF after 4 months per oral bisphosphonate followed by denosumab 4 years. LS T-score + 0.8, total hip −1.7. Delayed fracture healing before TPTD.
Mizuno et al. (2023)	79, male	TPTD 20 μg/day	24	Not noted	No fracture during therapy	ALP increased TRAP increased	LS T-score − 3.7 Alendronate 7 years → TPTD 2 years → minodronate 3 years Bone biopsy

AFF, atypical femoral fracture; ALP, alkaline phosphatase; BALP, bone-specific alkaline phosphatase; aBMD, areal bone mineral density; CKD, chronic kidney disease; CTX, C-terminal telopeptide of type I collagen; FN, femoral neck; HPP, hypophosphatasia; INTP and NTX, N-terminal telopeptide of type I collagen; LS, Lumbar spine; PINP, intact N-terminal propeptide of type I procollagen; PTH, parathyroid hormone; TPTD, teriparatide; TRAP, tartrate-resistant acid phosphatase.

The present case report describes a lifetime follow-up (to 65 years of age) of a woman diagnosed with HPP later in life, but with early-life symptoms compatible with childhood HPP and severe bone metabolic symptoms in adult life. She was thoroughly evaluated including mutation analysis of the *ALPL* gene, molecular modeling of the found variants, BALP isoform analysis, bone histomorphometry, bone densitometry, kidney biopsy, radiological and biochemical response to TPTD therapy for 9 months when she was 50 years old. This case was complicated by progressive chronic kidney disease (CKD), and she had to undergo a kidney transplantation at the age of 58 years with concerns for aggravation of the bone and mineral metabolic abnormalities.

## 2 Material and methods

### 2.1 *ALPL* sequencing and molecular modeling

Genomic DNA was extracted from blood leukocytes. All coding exons (no. 2–12) and adjacent mRNA splice sites of *ALPL* were analyzed for variants using methods reported elsewhere (Mumm et al., 2002).

The three-dimensional structure showing the patient's missense variants of *ALPL* affecting TNALP was based on the crystal structure of human TNALP reported in the Protein Data Base with identification number 7YIV (Yu et al., 2023). The dimer structure was compared to a previously reported Alphafold2 model of human TNALP (Jumper et al., 2021; Varadi et al., 2022; Atanasova et al., 2024), since the crystal structure was presented as an octamer (Yu et al., 2023). Both dimer structures were found to be near identical when analyzed with the superimposition modeling in PyMOL v2.5 (Schrödinger, LLC. 2010).

### 2.2 Biochemical measurements

Serum BALP was assayed with the Ostase® BAP enzyme-linked immunosorbent assay (ELISA) (Immunodiagnostic Systems Ltd., Boldon, UK), which has a reported cross-reactivity with liver ALP between 7 and 18 % (Magnusson et al., 2002b). This BALP immunoassay is not able to distinguish individual BALP isoforms and has different immunoreactivity properties towards these isoforms (Magnusson et al., 2002b). The reference interval for healthy individuals is 5–22 μg/L (Panigrahi et al., 1994).

The serum BALP isoforms B/I, B1 and B2 were determined by a previously described HPLC method (Magnusson et al., 1992; Magnusson et al., 1993). The BALP isoform reference intervals for healthy individuals are: B/I, 0.04–0.17 μkat/L; B1, 0.20–0.62 μkat/L; and B2, 0.34–1.69 μkat/L (Magnusson et al., 2001).

Serum routine clinical chemistry analyses were performed at the Department of Clinical Chemistry (Swedac accredited no. 1886), Karolinska University Hospital, Sweden.

### 2.3 Bone mineral density

Areal bone mineral density (aBMD) for total body, hip, and lumbar spine was assessed by dual-energy X-ray absorptiometry (DXA) using both the GE Healthcare Lunar DXA (GE Lunar Corp., Madison, WI, USA) and Hologic DXA systems (Hologic Discovery A, MA, USA) over the time span of this case report. All measurements were performed at the Department of Radiology at Karolinska University Hospital, Solna, Sweden.

### 2.4 Bone histomorphometry

A 2–10–2-6 day tetracycline double labeling was given prior to the first bone biopsy. That is, oral tetracycline hydrochloride 250 mg four times daily for 2 days, followed by a 10-day tetracycline-free period and another 2 days with tetracycline four times daily, and then a 6-day tetracycline-free period. The transiliac bone biopsy was obtained at age 44 years from a location 2 cm below the iliac crest and 2 cm posterior to the anterior-superior iliac spine using a trephine drill. The bone biopsy was immersed in 70 % ethanol for fixation followed by dehydration and embedded undecalcified in methyl methacrylate (Erben, 1997).

A second iliac crest bone biopsy was obtained at 55 years of age, 5 years after ending the TPTD therapy, to evaluate the degree of bone metabolic disturbance and the possibility of an additional period with TPTD therapy. After a 2–10–2-5 day double labeling with tetracycline 500 mg twice daily, a 3.5 × 20 mm bone core was retrieved by vertical biopsy from 2 cm posterior to the anterior superior iliac spine, using a Yamshidi bone biopsy needle and was processed as above.

After embedding, sections of 7 μm were cut from two levels with a distance of 100 μm using a Jung microtome model K (R. Jung GmbH, Heidelberg, Germany) equipped with a tungsten carbide knife. Sections were stained with Masson Goldner trichrome for light microscopy or mounted unstained for epifluorescent microscopy.

Histomorphometry of both bone biopsies was performed using the principle of vertical sections (Baddeley et al., 1986) using a Nikon ECLIPSE 80i light microscope (Nikon, Tokyo, Japan) equipped with a Prior Proscan 11 motorized specimen stage (Prior Scientific, Inc., Rockland, MA, USA) and a Olympus DP72 digital video camera (Olympus, Tokyo, Japan) connected to a PC running the newCAST interactive stereology software (Visiopharm, Hoersholm, Denmark). Measurements of bone volume per tissue volume (magnification ×195), osteoid surface per bone surface (×391), erosion surface per bone surface (×391), single- and double-labelled surface per bone surface (×391), mineralizing surface per bone surface (×391), and mineral apposition rate (×784) were in accordance with the guidelines provided by the American Society for Bone and Mineral Research (Dempster et al., 2013). CVs were 1.7 % for bone volume per tissue volume, 1.1 % for osteoid surface per bone surface, and 8.3 % for erosion surface per bone surface.

## 3 Results

### 3.1 Case history before endocrine referral, age 1–39 years

**Fig. 1:**
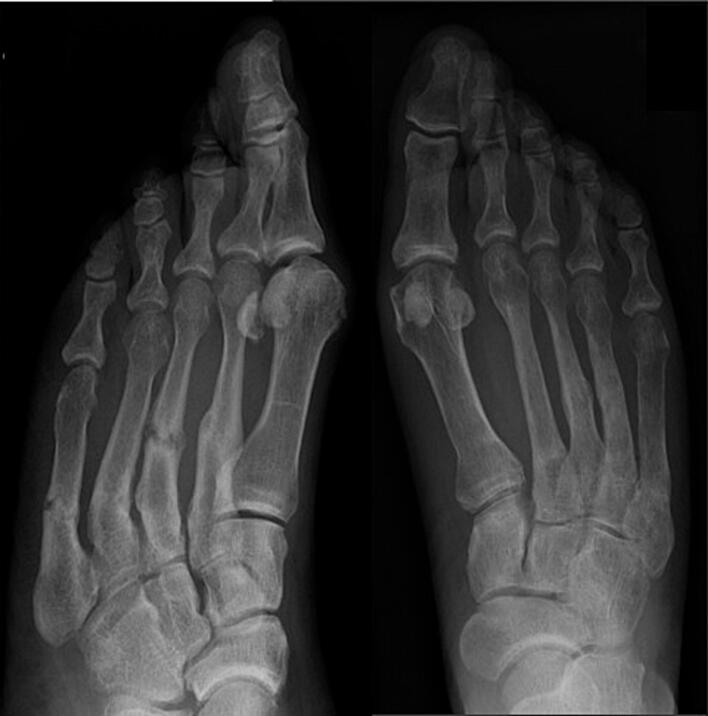
Radiographic image of metatarsal bone fractures II-V bilaterally.

The patient was 39 years old when referred to the Department of Endocrinology, Karolinska University Hospital, Sweden, for recurring metatarsal fractures. She worked part-time as an assistant nurse. Her history revealed that she had craniosynostosis surgery during infancy and early loss of deciduous teeth. There was no failure to thrive or abnormal height growth. At 10 years of age, hematuria was attributed to a urinary tract infection and treated with antibiotics but followed by persistent proteinuria. At age 25 years she was diagnosed with hypertension. She was two-parous at 25 years and 35 years of age without complications except for metatarsal fractures during her first pregnancy. She delivered two healthy children. She had recurrent pain in both feet since age 27 years, and at the age of 28 years, radiographs confirmed metatarsal fractures II–V bilaterally (Fig. 1).

**Fig. 2:**
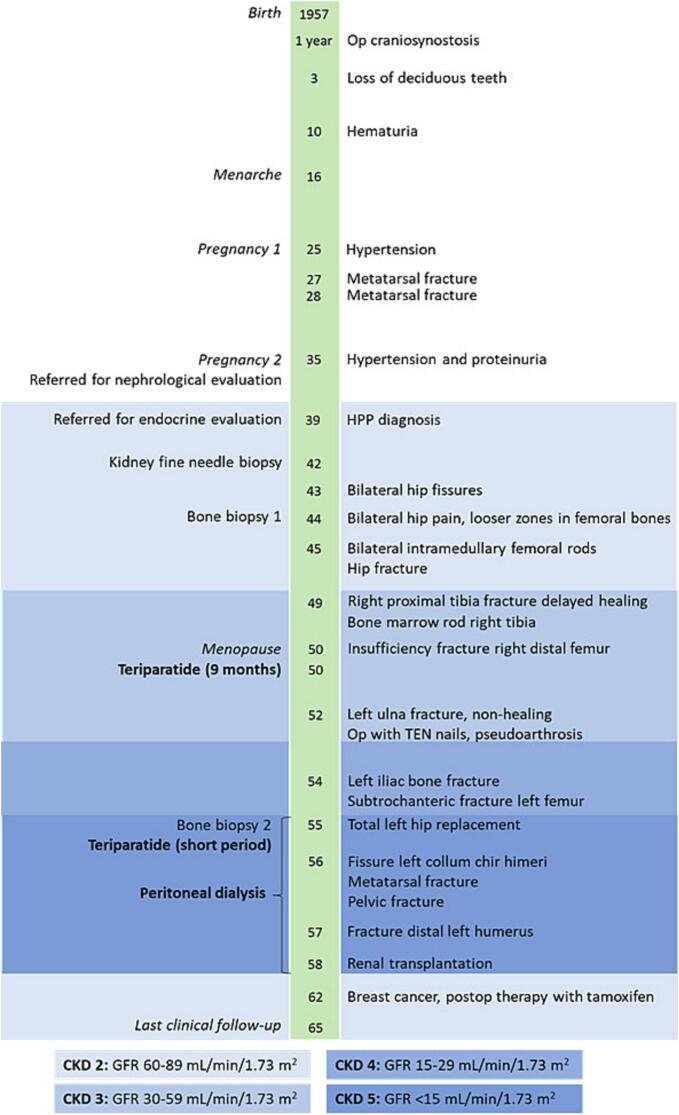
Timeline for the patient's clinical course from birth to 65 years of age.

A timeline including CKD stages following the degree of kidney deterioration (Levin and Stevens, 2014), from birth to the last clinical follow-up at 65 years of age, is presented in Fig. 2.

### 3.2 Age 39–50 years

**Fig. 3:**
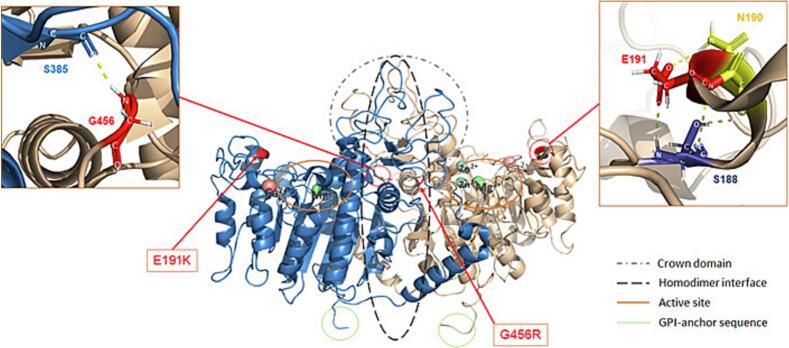
A three-dimensional structure of TNALP showing the patient's two missense mutations. Ions: green = magnesium, turquoise = zinc, pink = calcium. Two missense mutations were found: exon 6, c.571G > A, p.Glu191Lys (E191K); and exon 12, c.1366G > A, p.Gly456Arg (G456R). Mutation G456R is located in the homodimer interface. The main chain of G456 interacts with the main chain of S385 located on the other monomer, which contributes for dimer formation (unclear if the mutations would lead to diminished polar interaction). The mutation E191K is located in a peripheral loop close to the active site. The main chain of E191 form polar interactions with the main chains of N190 and S188, possibly stabilizing the loop around the active site.

Initial endocrine evaluation, at age 39 years, serum total ALP activity was below the detection limit of 0.3 μkat/L (18 U/L) and serum BALP (by ELISA) was below the detection limit of 0.3 μg/L. The diagnosis of HPP was confirmed by genetic analysis, which demonstrated compound heterozygosity for 2 missense variants: exon 6, c.571G > A, p.Glu191Lys (pathogenic); and exon 12, c.1366G > A, p.Gly456Arg (likely pathogenic) (Farman et al., 2024). The parents were not available for study, so we could not prove that the variants were in trans. The functional effects of the found variants, in relation to the different TNALP molecular domains, are presented in Fig. 3. The patient fulfilled the diagnostic criteria for adult HPP previously suggested by Berkseth et al. (2013), although symptoms occurred at an early age.

HPLC analysis of the BALP isoforms revealed exceptionally low activities of all 3 BALP isoforms, i.e., B/I 0.01 μkat/L (0.6 U/L), B1 0.04 μkat/L (2.4 U/L), and B2 0.10 μkat/L (6.0 U/L). Serum ionized calcium, phosphate, magnesium, PTH, 25-hydroxyvitamin D, 1,25-dihydroxyvitamin D, creatinine, urinary calcium and markers of bone turnover (serum osteocalcin, urinary hydroxyproline and deoxypyridinoline) were all within the reference intervals for healthy individuals.

#### 3.2.1 Fractures and aBMD

**Fig. 4:**
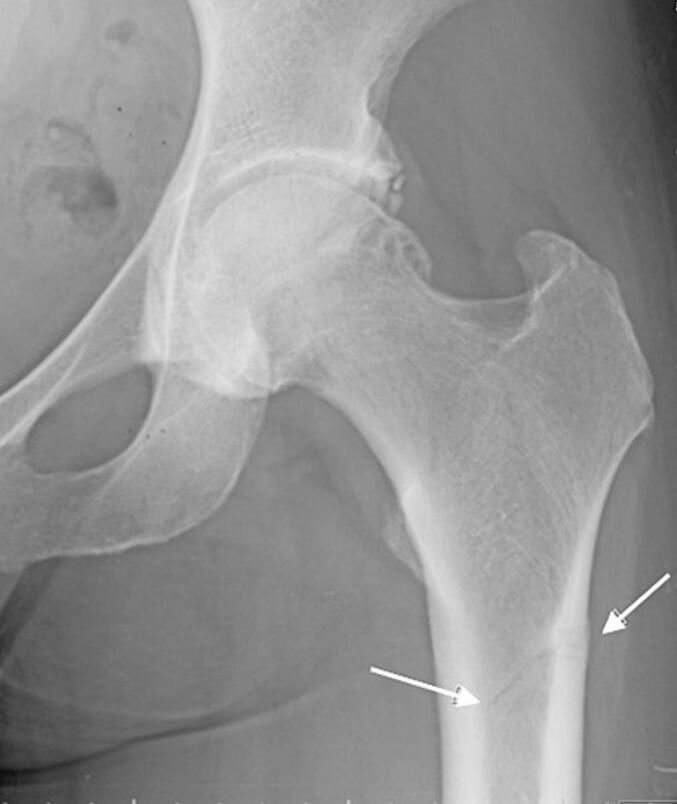
Fissure fracture of the left hip.

At 43 years of age, hip pain was evaluated by magnetic resonance tomography, which showed bilateral hip fissures that were handled conservatively without surgery (Fig. 4). However, during a minor fall at age 45 years, she dislocated the right fissure and underwent surgery with bilateral intramedullary rodding. At age 49 years, spontaneous fracture of her right proximal tibia had delayed healing and then treated with rod placement. At age 50 years, she became menopausal and had an insufficiency fracture of the right distal femur bone that occurred during low intensity exercise. The CKD progressed and because of recurring low energy fractures it was decided to start treatment with TPTD.

Total body aBMD (by Lunar) at age 40 years and 43 years were 1.072 g/cm^2^ (T-score − 0.7) and 1.088 g/cm^2^ (T-score − 0.5), respectively (Table 2). Total hip aBMD T-score values at ages 38, 40 and 43 years were − 1.0, −0.2 and − 0.9, respectively. Total body and hip aBMD were not assessed after age 45 years because of osteosynthesis material. The lumbar spine aBMD values (by Lunar) were normal between the ages 40 to 49 years: T-score L1–L4 varied between −0.4 to 0.9.

**Table 2:** Sequential lumbar spine aBMD.

Age (years) (Lunar or Hologic instrumentation)	L1–L4 (g/cm^2^)	L1–L4 (*Z*-score)	L1–L4 (T-score)	L3–L4 (g/cm^2^)	L3–L4 (Z-score)	L3–L4 (T-score)
40 (Lunar)	1.155	0.0	−0.4	NA	NA	NA
43 (Lunar)	1.177	0.2	−0.2	NA	NA	NA
48 (Lunar)	1.287	1.6	0.9	1.280	1.3	0.7
49 (Lunar)	1.259	1.2	0.7	1.256	1.0	0.5
51 (Lunar)	1.228	1.2	0.4	1.228	1.0	0.2
54 (Lunar)	1.125	0.7	−0.5	1.088	0.2	−0.9
54 (Hologic)	0.954	0.2	−0.8	0.916	−0.3	−1.7
55 (Hologic)	0.900	−0.2	−1.3	0.867	−1.0	−2.1
57 (Hologic)	0.818	−0.9	−2.1	0.763	−1.8	−3.1
58 (Hologic)	0.913	0.1	−1.2	0.865	−0.8	−2.1
61 (Hologic)	0.926	0.4	−1.1	0.904	−0.2	−1.8

aBMD, areal bone mineral density; NA, not available.

#### 3.2.2 Bone histomorphometry

**Fig. 5:**
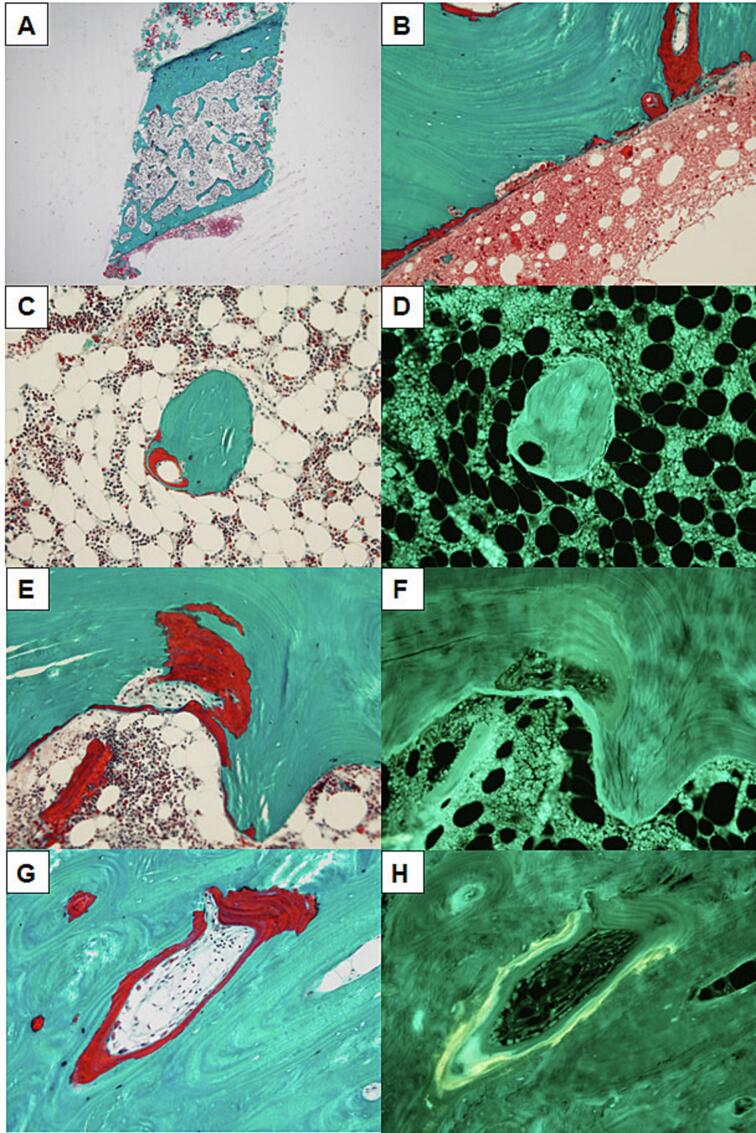
Bone histomorphometry from a transiliac bone biopsy at 44 years of age, CKD stage 2. (A) Cancellous and cortical bone tissue is lamellar and present in lower amounts and less interconnected than expected from age. (B) The bone erosion surface was low, but erosion pits interrupting the lamellar structure were seen at the periosteal side of the cortex. (C and E) Traces of focally high osteoclast activity were demonstrated by undermined osteoid seams in the cancellous bone. (C, E and G) The osteoid surface was low and the osteoid seams thin and predominantly without osteoblasts. (C and E) Foci of thick lamellar and non-lamellar osteoid seams were embedded within bone and showed an irregular interface between osteoid and bone tissue. (D, F and H) Tetracycline double labels were demonstrated only in formative sites with osteoblasts present. No fibrosis was seen in the bone marrow.

A transiliac biopsy was obtained at the age of 44 years, 6 years before starting therapy with TPTD (Table 3, Fig. 5). Cancellous bone was lamellar, present in lower amounts and less interconnected than expected from age. Bone turnover was low as indicated by low resorption surfaces, although high osteoclast activity was seen focally. Low osteoid surface and thin osteoid seams, predominantly without osteoblasts, aligned with low bone turnover. Foci with thick osteoid seams, not covered by osteoblasts, and absence of tetracycline labels indicated disturbed mineralization. Tetracycline double labels were demonstrated only in formative sites with osteoblasts present. No fibrosis was seen in the bone marrow. Taken together, the histological findings were consistent with HPP and there were no signs of hyperparathyroidism.

**Table 3:** Bone histomorphometric parameters.

	Biopsy 1 Age 44 years	Biopsy 2 Age 55 years
Bone volume per tissue volume (%)	12.5	17.2
Osteoid surface per bone surface (%)	17.4	43.5
Erosion surface per bone surface (%)	16.3	14.8
Single-labelled surface per bone surface (%)	0	0
Double-labelled surface per bone surface (%)	0	0
Mineralizing surface per bone surface (%)	0	0
Mineral apposition rate (μm/day)	NA	NA

NA, not available.

#### 3.2.3 CKD

Due to progressive deterioration of kidney function, a fine-needle kidney biopsy at age 42 years was consistent with focal segmental glomerular sclerosis. The biopsy also showed nonspecific interstitial calcification, but it was not classified as nephrocalcinosis. Her history of hematuria and proteinuria during childhood, along with the development of hypertension at age 25 years, suggests early-onset kidney disease. There was no reported history of symptomatic nephrolithiasis.

### 3.3 TPTD treatment at 50 years of age

TPTD is a commercially available recombinant PTH fragment 1–34 for the treatment of postmenopausal osteoporosis and high risk of imminent fractures. Injected daily, PTH is anabolic for bone stimulating osteoblasts and increasing bone turnover and several bone metabolic markers (Neer et al., 2001). At age 50 years, in CKD stage 3b (estimated glomerular filtration rate (eGFR) 34 mL/min/1.73 m^2^), she received a 9-month course of TPTD (Forsteo®, Eli Lilly, Indianapolis, IN, USA) 20 μg/day, interrupted by a 2-week break. No symptomatic fissures or fractures occurred during the TPTD therapy and less bone pain was noticed, while the patient experienced no subjective adverse effects. Serial determination of aBMD, using the Lunar DXA, revealed no effect of TPTD treatment at the lumbar spine L1–L4 (Table 2). Hip aBMD was not possible to measure due to osteosynthesis material.

Serum ionized calcium increased from 1.30 mmol/L to 1.44 mmol/L (reference interval 1.15–1.33 mmol/L) after 3 months of TPTD therapy and remained increased throughout the 9 months of therapy, returning to baseline levels 3 months after ending the treatment. Serum phosphate similarly increased from 1.5 mmol/L to 2.3 mmol/L (reference interval 0.8–1.5 mmol/L) after 3 months of TPTD therapy and returned to baseline levels after treatment. TPTD therapy was stopped after 9 months due to hypercalcemia, hyperphosphatemia, and progression of her CKD to stage 3b (eGFR 33 mL/min/1.73 m^2^). During TPTD treatment, serum PTH increased from 30 ng/L to 64 ng/L (reference interval 15–65 ng/L). Serum total ALP and BALP (measured by ELISA) was undetectable throughout the TPTD therapy; however, increased activities were found for all BALP isoforms B/I, B1 and B2. The B2 isoform was 0.42 μkat/L (25 U/L) after 2.5 months of therapy, which was within the reference interval 0.34–1.69 μkat/L (20–101 U/L).

### 3.4 Age 50–65 years

#### 3.4.1 Fractures and aBMD

At age 52 years, she fractured her left ulna during minor trauma and had surgery with intramedullary fixation after a few weeks due to non-healing. Two years later, she experienced a left subtrochanteric and iliac bone fracture, which was treated conservatively. At age 55 years, a broken left femoral intramedullary rod led to total hip replacement.

Because of the decreased bone pain and absence of new fractures during the previous TPTD therapy, a new trial was undertaken, but was interrupted after only 1 month due to an increase of serum ionized calcium to 1.43 mmol/L. The bone resorption inhibitors bisphosphonates and denosumab were considered contraindicated since bone histomorphometry had shown osteomalacia.

DXA results are summarized in Table 2. Lumbar spine aBMD measurements were limited to L3–L4 from age 55 years because of irregular values indicating falsely high values for L1–L2. Lumbar spine L3–L4 aBMD T-score values (by Hologic) decreased from −1.7 at age 54 years, to −3.1 at age 57 years, but then increased to −1.8 at age 61 years.

#### 3.4.2 Bone histomorphometry

**Fig. 6:**
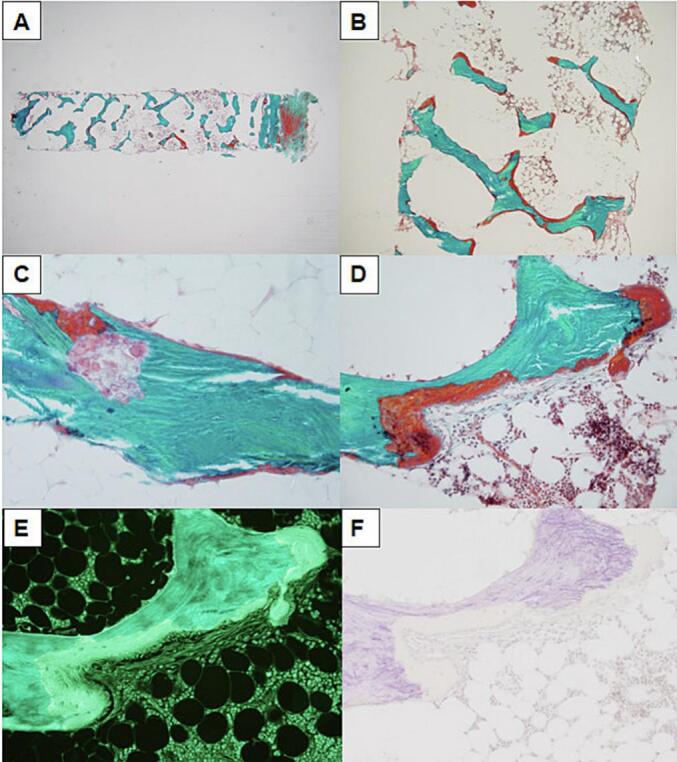
Bone histomorphometry from a vertical iliac crest bone biopsy at 55 years of age, CKD stage 5. (A) Cancellous and cortical bone tissue is lamellar and present in lower amounts and less interconnected than expected from age. (B) The osteoid surface was high. The osteoid seams were thicker than normal and predominantly without osteoblasts. Some osteoid seams were located at bone surfaces without prior bone resorption. (C) The bone erosion surface was low. Traces of focally high osteoclast activity were demonstrated by erosion pits embedded in the cancellous bone tissue. (D) Foci of accumulated lamellar and non-lamellar osteoid seams with an irregular interface between osteoid and bone tissue were found. Fibrosis was not a typical presentation in the bone marrow; however, local fibrosis was seen in the bone marrow close to this site of remodeling activity. (E) Tetracycline labeling was weak and only single labels were demonstrated at formative sites with osteoblasts present. (F) Staining for ALP was negative.

A second bone biopsy was performed when she was in CKD stage 5, at the age of 55 years, 5 years after ending the first course of TPTD therapy, to evaluate the degree of bone metabolic disturbance and the possibility to initiate a new TPTD therapy (Table 3, Fig. 6). Bone turnover was low as indicated by low resorption surfaces, however, a focus was observed that may be tunneling cut across. Osteoid surfaces and osteoid thickness were increased indicating disturbed mineralization while bone volume was not compromised, resembling a histological picture of osteomalacia. In comparison with the first bone biopsy 11 years earlier and before start of TPTD treatment, in the second biopsy 5 years after TPTD therapy there was a higher proportion of osteoid surfaces laid on the bone surface without significant prior resorption and there were fewer osteoblasts on the bone surface.

#### 3.4.3 End-stage kidney disease and kidney transplantation

At age 55 years, her kidney function deteriorated to CKD stage G5 with an eGFR of <15 mL/min/1.73 m^2^, and peritoneal dialysis was started. Successful kidney transplantation occurred at age 58 years. She was prescribed a reduced cortisone schedule to reduce the risk of worsening her bone and mineral metabolic status due to HPP. Following the kidney transplantation, her well-being improved and she had less bone pain and no new fractures. Lumbar spine BMD (Hologic L3–L4) increased with 13 % and an additional 4.6 % (T-score − 1.8), 1 and 3 years after the kidney transplantation, respectively.

#### 3.4.4 Last clinical follow-up

At age 62 years, she was diagnosed and operated for estrogen receptor positive breast cancer with postoperative therapy with tamoxifen, a selective estrogen receptor modulator drug chosen because of less risk of negative effects on bone than aromatase inhibitor treatment. Most biochemical measures normalized at 65 years of age (last clinical follow-up): eGFR 79 mL/min/1.73 m^2^, phosphate 1.3 mmol/L, ionized calcium 1.26 mmol/L and PTH 59 ng/L, but serum total ALP remained below the detection limit of 0.3 μkat/L (18 U/L).

## 4 Discussion

This patient illustrates important clinical features of HPP complicated by CKD progressing to end-stage renal disease, requiring dialysis and kidney transplantation. The low awareness and limited knowledge of HPP in the 1950s to 1980s among healthcare professionals contributed likely to the delayed diagnosis of HPP in this woman. The awareness of HPP has improved considerably over the years, particularly among pediatric clinicians. Another improvement over the years, in clinical laboratory medicine, is the use of accurate pediatric age- and sex-specific reference intervals for serum total ALP and BALP levels (Whyte et al., 2018).

Patients with HPP commonly have fractures of metatarsal bones and femoral diaphysis, and less often vertebral fractures in comparison with osteoporotic patients (Desborough et al., 2021). Until the age of 54 years (before peritoneal dialysis), this patient had normal aBMD lumbar spine values, but still several recurring fractures in other sites. Hence, aBMD in adult HPP patients is not systematically low. A recent study, comprising 110 individuals with adult HPP, even found that higher aBMD lumbar spine values were associated with more serious HPP forms and higher fracture risk (Genest et al., 2021). In this study, the DXA assessments were initially performed using the Lunar system and subsequently with the Hologic system, which we acknowledge as a methodological limitation. The reported *Z*-scores and T-scores are system-specific and based on the respective manufacturer's reference data. Notably, no universally accepted gold standard DXA machine exists. While the use of two DXA systems introduces some variability, this reflects real-world clinical practice where multiple systems are in use.

High-resolution peripheral quantitative computed tomography would have provided data on trabecular and cortical bone structures, volumetric bone density and cortical porosity. We acknowledge the limitation that data from volumetric imaging techniques are not available, particularly given the coexistence of HPP and end-stage renal disease, both of which impair skeletal integrity independently of areal BMD.

Her Glu191Lys TNALP variant has been found in 31 % of mild HPP and is the most frequent variant with Caucasian origin (Hérasse et al., 2002). Several reports have shown that the heterozygous Glu191Lys variant has a 88 % residual ALP activity (Henthorn et al., 1992; Zurutuza et al., 1999; Taillandier et al., 2005) and the variant is derived from a common ancestor from Western Europe (Hérasse et al., 2002). The variant is located near the TNALP active site and is proposed to be involved in guiding the substrate to the active site (Mornet et al., 2001). However, heterozygous carriers for this variant have been described as symptom-free with normal ALP activities (Taillandier et al., 2005; Fauvert et al., 2009). The benign nature of the heterozygous Glu191Lys variant might explain its prevalence in Europe and America (Hérasse et al., 2002). The Glu191Lys variant, with one or more other variants, is common in most of the severe childhood and adult HPP patients, suggesting compound heterozygosity or a dominant-negative effect of other more severe variants (Fauvert et al., 2009). The other detected variant, Gly456Arg, has previously been reported in one case of neonatal HPP with a heterozygous genotype in Japan (Ozono et al., 1996; Yokoi et al., 2019) and in severe adult HPP in a Spanish population (Riancho-Zarrabeitia et al., 2016). Gly456Arg is located in the dimer interface and dimerization is required for maintaining stable protein fold and enzymatic activity (Mornet et al., 2001). Expression studies have identified a dominant negative effect of this variant with a residual ALP activity of 2 % (del Angel et al., 2020).

Four out of the 13 case reports studying TPTD therapy in 18 patients with adult HPP comprise data on bone histomorphometry (Table 1) (Gagnon et al., 2010; Cundy et al., 2015; Schmidt et al., 2019; Mizuno et al., 2023). In the case report presented by Gagnon et al. (2010), the first bone biopsy before TPTD therapy revealed osteomalacia and a second biopsy, after TPTD therapy, showed increased numbers of osteoblasts and a higher amount of osteoid volume, but persistent impaired mineralization. Cundy et al. (2015) performed three bone biopsies in a male adult with HPP and CKD. The first bone biopsy, taken 3 months after alendronate treatment for 21 months and before TPTD, showed marked osteomalacia with low osteoblast numbers. A second bone biopsy, taken directly after 6 months of TPTD, showed improved osteoid and trabecular parameters. Noticeably, total ALP increased by 60 % and BALP by 250 % during TPTD treatment. The third bone biopsy, taken 18 months after kidney transplantation, showed a return to marked hyperosteoidosis, but in contrast to the first biopsy a large proportion of the osteoid was covered by osteoblasts and the mineral apposition rate was normalized. Schmidt et al. (2019) presented bone histomorphometry data in one individual with CKD stage 3 and adult HPP 8 weeks after onset of TPTD therapy. The bone biopsy showed increased osteoid volume and severe osteomalacia. The recent report by Mizuno et al. (2023) presents data from a patient that was treated with alendronate for 7 years followed by TPTD for 2 years, and sequential treatment with minodronate for several years. A bone biopsy was taken 1 year into the minodronate treatment (i.e., 1 year after ending the TPTD therapy), which showed adynamic trabecular bone, increased cortical bone formation, and tetracycline labeling only in cortical bone.

The bone histomorphometric assessments in the present case revealed signs of HPP with osteomalacia but no obvious signs of hyperparathyroidism in either of the two biopsies when the patient was in CKD stage 2 or stage 5. CKD progression may have contributed to the more pronounced mineralization defect in the second bone biopsy. In the latter biopsy, there was osteoid surface with no considerable previous resorption, possibly connected to previous teriparatide therapy even if given 5 years earlier. It has previously been stated that osteomalacia is present in HPP except in odontohypophosphatasia and that hyperparathyroidism is usually not found in HPP (Whyte, 2016). In contrast to the often massive increase in total ALP or BALP after TPTD treatment in the published cases with bone histomorphometry, the here reported patient showed no increase of total ALP or BALP, while some increase of all BALP isoforms was noted, indicating that the patient has a more severe variant of HPP.

A few cases have been published with HPP requiring dialysis (Cundy et al., 2015; Whyte et al., 2013; Remde et al., 2017) and kidney transplantation (Cundy et al., 2015). In one of these cases, hemodialysis was required due to severe acute hypercalcemia after traumatic fractures and immobilization (Whyte et al., 2013). Kidney biopsies from adult patients with HPP have shown focal glomerulosclerosis or IgA nephritis (Cundy et al., 2015). Individuals with HPP can be more prone to kidney damage due to higher serum calcium and phosphate levels and have also higher levels of pyrophosphate due to the ALP deficiency (Dahir et al., 2022). Kidneys contain generally high amounts ALP in the brush border epithelia of the proximal tubules; however, the direct significance and biological function of kidney ALP has not been elucidated (Haarhaus et al., 2017).

Kidney transplantation may trigger adverse health effects for the skeleton in patients with HPP due to the global critical situation, operation trauma, bone metabolic disturbances, and combinations of immunosuppressive therapy, generally comprising glucocorticoids. This could be considered as a hazardous situation for HPP patients motivating modified immunosuppressive schedules with lower doses of cortisone or steroid free schedules. The present case had no new fractures and less bone pain during the 7 years after her kidney transplantation and experienced a remarkable increase in BMD during the first and second year after transplantation. In the absence of an additional bone biopsy after kidney transplantation, causes for this improvement remain unidentified, but contributions by improved vitamin D metabolism or reduced fibroblast growth factor-23 levels could be hypothesized (Kanaan et al., 2010; Bilha et al., 2020). Another published case of an adult with HPP and CKD also did better after the kidney transplantation, suggesting that in HPP patients, CKD on dialysis may be more deleterious to the skeleton than the challenge of undergoing a kidney transplantation (Cundy et al., 2015).

The present case had less bone pain, improved fracture healing and had no fractures during the 9-month period of TPTD therapy. Lumbar spine BMD decreased during the same period, which is a similar finding reported in another case report of TPTD therapy (Whyte et al., 2007). Most, but not all reports have noted increased bone turnover markers during TPTD therapy but the results have often not been sustainable (Table 1). Reported effects of TPTD therapy on aBMD have been variable; however, fracture healing and bone pain improved in most HPP cases. The reported variable effects of TPTD therapy may be associated with functional differences of the found *ALPL* variants (Gagnon et al., 2010; Laroche, 2012; Mornet et al., 2021).

Inhibitors of bone resorption, such as bisphosphonates and denosumab (Warren et al., 2021; McKiernan et al., 2014), decrease the overall bone remodeling rate and can be expected to worsen the mineral metabolic state since HPP patients are at risk of deficient mineralization and osteomalacia due to their ALP deficiency. Analysis of serum total ALP or, even better, BALP, should be included in evaluation of individuals with fragility fractures to exclude HPP before contemplating osteoporosis medication.

As an alternative to the anabolic PTH analogue TPTD, some individuals with adult HPP have been treated with asfotase alfa with seemingly positive results for symptoms and biochemistry (Kishnani et al., 2019). One case has been published where an adult on hemodialysis and with childhood HPP was treated for 13 months with asfotase alfa that improved skeletal pain and fracture healing (Remde et al., 2017). Terminal kidney failure can cause complex bone metabolic disturbances such as CKD–mineral bone disorder, osteitis fibrosa cystica, osteomalacia and adynamic bone disease (Hu et al., 2022); however, this is not well studied in individuals with HPP. Before initiating off-label use in individuals with adult HPP and advanced CKD, characterization of the bone mineral metabolic status is recommended.

## 5 Conclusions

This lifetime follow-up over 65 years of a woman with HPP and advanced CKD shows that she improved clinically during 9 months of TPTD treatment. All BALP isoforms increased during TPTD treatment indicating improved mineralization. TPTD could be an alternative for temporary treatment in adult HPP with bone pain and delayed fracture healing. The kidney transplantation had a beneficial outcome for her bone health since no fractures or severe pain were observed 7 years after kidney transplantation.

## CRediT authorship contribution statement

**Maria Sääf:** Writing – review & editing, Writing – original draft, Project administration, Methodology, Investigation, Formal analysis, Data curation, Conceptualization. **Sigridur Björnsdottir:** Writing – review & editing, Writing – original draft, Project administration, Methodology, Investigation, Formal analysis, Data curation, Conceptualization. **Mathias Haarhaus:** Writing – review & editing, Validation, Resources, Methodology, Investigation, Formal analysis. **Ellen-Margrethe Hauge:** Writing – review & editing, Visualization, Validation, Resources, Methodology, Investigation, Formal analysis. **Diana Atanasova:** Writing – review & editing, Writing – original draft, Visualization, Validation, Methodology, Investigation, Formal analysis, Data curation. **Per Magnusson:** Writing – review & editing, Writing – original draft, Visualization, Validation, Supervision, Resources, Project administration, Methodology, Investigation, Funding acquisition, Formal analysis, Data curation, Conceptualization.

## Declaration of Generative AI and AI-assisted technologies in the writing process

During the preparation of this work the authors used Microsoft Copilot in order to improve sentence structure in the title and parts of Introduction and Discussion. After using this tool/service, the authors reviewed and edited the content as needed and take full responsibility for the content of the published article.

## Funding

Open Access funding provided by Linköping University Library. This work was supported by grants from the 10.13039/501100004359Swedish Research Council and ALF grants from Region Östergötland.

## Declaration of competing interest

The authors declare that they have no known competing financial interests or personal relationships that could have appeared to influence the work reported in this paper.

## Data Availability

Data will be made available on request.
